# IRE1α mediates PKR activation in response to *Chlamydia trachomatis* infection

**DOI:** 10.1016/j.micinf.2016.03.010

**Published:** 2016

**Authors:** Steve J. Webster, Lou Ellis, Louise M. O'Brien, Beatrice Tyrrell, Timothy J. Fitzmaurice, Matthew J. Elder, Simon Clare, Ronnie Chee, J.S. Hill Gaston, Jane C. Goodall

**Affiliations:** a Rheumatology Research Group, Department of Medicine, University of Cambridge, UK; b Wellcome Trust Sanger Institute, Wellcome Trust Genome Campus, Hinxton, UK; c Department of Immunology, Royal Free Hospital, London, UK

**Keywords:** PKR, ER stress, *Chlamydia*

## Abstract

Protein kinase RNA activated (PKR) is a crucial mediator of anti-viral responses but is reported to be activated by multiple non-viral stimuli. However, mechanisms underlying PKR activation, particularly in response to bacterial infection, remain poorly understood. We have investigated mechanisms of PKR activation in human primary monocyte-derived dendritic cells in response to infection by *Chlamydia trachomatis*. Infection resulted in potent activation of PKR that was dependent on TLR4 and MyD88 signalling. NADPH oxidase was dispensable for activation of PKR as cells from chronic granulomatous disease (CGD) patients, or mice that lack NADPH oxidase activity, had equivalent or elevated PKR activation. Significantly, stimulation of cells with endoplasmic reticulum (ER) stress-inducing agents resulted in potent activation of PKR that was blocked by an inhibitor of IRE1α RNAse activity. Crucially, infection resulted in robust IRE1α RNAse activity that was dependent on TLR4 signalling and inhibition of IRE1α RNAse activity prevented PKR activation. Finally, we demonstrate that TLR4/IRE1α mediated PKR activation is required for the enhancement of interferon-β production following *C. trachomatis* infection. Thus, we provide evidence of a novel mechanism of PKR activation requiring ER stress signalling that occurs as a consequence of TLR4 stimulation during bacterial infection and contributes to inflammatory responses.

## 1 Introduction

Protein kinase RNA activated (PKR) was originally identified as a cytosolic kinase that was activated by double stranded RNA (dsRNA) that could terminate protein translation by acting as an eIF2α kinase [1], [2], [3]. PKR activation occurs as a consequence of auto -phosphorylation at several serine and threonine residues following the binding of dsRNA within the N′ terminus and phosphorylation of Thr451 within the activatory domain is critical for PKR function [4]. The role of PKR during viral infection is well documented. However, PKR is also activated during Toll Like Receptor (TLR) signalling, independent of dsRNA, and regulates inflammatory responses and cell death [5], [6]. Additionally, PKR has been reported to be necessary for NLRP3 and NLRC4 inflammasome activation [7] although these findings have proved controversial [8]. Furthermore, sterile agonists such as cholesterol, palmitic acid [9], [10] and the endoplasmic reticulum (ER) stress-inducing agents tunicamycin and thapsigargin can all induce PKR activation [10], [11]. These data indicate that in addition to its function during viral infection, PKR also responds to a variety of stimuli such as bacterial infection, and to metabolic or ER stress. However, despite overwhelming evidence that PKR is activated by a wide range of stimuli, the mechanism(s) of how this occurs, particularly as a result of TLR stimulation and bacterial infection, is poorly understood. We have investigated mechanisms of PKR activation in response to a common intracellular bacterial infection; *Chlamydia trachomatis*, and the role that TLR4, ER stress and the NADPH oxidase system play in the process.

## 2 Methods

### 2.1 Reagents

Ultra pure LPS (*Escherichia coli*) and Poly I:C were obtained from Invivogen (France), peptidoglycan (*Bacillus subtilis*) was obtained from Sigma (U.K.), and curdlan (*Alcaligenes faecalis*) was obtained from Wako (U.S.A). Anti-phospho Thr451-PKR was obtained from Millipore (U.K.), anti-PKR (D20) from SantaCruz (U.S.A) and anti-actin from Abcam (U.K.). The IRE1α inhibitor (4μ8c) was obtained from Tocris (U.K.), the PKR inhibitor (C16) from Calbiochem (Germany) and the PERK inhibitor (GSK PERK inhibitor-D3) from Toronto Research Chemicals (Canada). Anti-TLR4 blocking antibody, inhibitory peptides for MyD88 and TRIF were all from Invivogen (France).

### 2.2 Cell culture

Human monocyte-derived dendritic cells (mDC) were cultured from monocytes obtained from apheresis transfusion cones (National transfusion service U.K.) by ficoll density centrifugation and positive CD14 selection using micro-beads (Miltenyi, U.K.) to achieve monocyte cultures that were >90% pure. Monocytes were cultured for 6-days in RPMI1640 containing 5% FCS, 20 ng/ml GM-CSF (Gibco, U.K.) and 4 ng/ml IL-4 (BD Pharmingen, U.K.). Murine bone marrow derived macrophages (BMDM) were isolated from the femurs of littermate wild type (*cybb*^+/+^ or *pkr*^+/+^), gp91 phox deficient (*cybb*^−/−^) or PKR deficient (*pkr*^−/−^) C57BL6 mice and cultured for 7-days in RPMI1640 containing 10% FCS and supplemented with 5% L929 conditioned media. Wild type (*gcn2*^+/+^) or GCN2 deficient (*gcn2*^−/−^) mouse embryonic fibroblasts (MEFs) were cultured in DMEM containing 10% FCS and supplemented with 55 μM β-mercaptoethanol.

### 2.3 Cell stimulations and infections

BMDM or mDC were harvested by scraping and plated at 5 × 10^5^ cells/well of a 24-well plate (Costar). Human mDC were stimulated for 4-h with either 1 μg/ml LPS, 10 μg/ml peptidoglycan (PGN), 25 μg/ml Poly I:C (PIC), or 100 μg/ml Curdlan (CUR). BMDM or human mDC were infected with *C. trachomatis* at a multiplicity of infection (MOI) of 20 (unless stated otherwise) for indicated times. Attenuated *C. trachomatis* was prepared by gamma irradiation or heat inactivation. Where inhibitors were used, cells were pre-treated at least 1 h prior to cell stimulation or infection with the exception of the MyD88 and TRIF inhibitory peptides that were used at least 4 h prior to stimulation or infection. MEFs were plated at 5 × 10^5^ cells/well of a 6-well plate (Corning) and infected with the murine pathogen *Chlamydia muridarum* or *C. trachomatis* at MOI = 10 followed by centrifugation at 2000 × G for 40 min to aid infectivity.

### 2.4 Preparation of cytoplasmic lysates for immunoblotting

BMDM and mDC were washed once in cold PBS. The cells were then lysed on ice in 300 μl of ice cold cytoplasmic lysis buffer (10 mM HEPES, 50 mM NaCl, 0.5 M Sucrose, 0.1 mM EDTA, 0.5% v/v Triton X-100, 10 mM Tetrasodium pyrophosphate, 17.5 mM β-glycerophosphate and one complete mini-protease inhibitor cocktail tablet). After lysis the cytoplasmic extract was frozen at −20 °C overnight before thawing to aid cell lysis. The lysate was then centrifuged 15,000 × G for 15 min at 4 °C and the supernatant retained. Protein quantification of the lysates was carried out by Bradford assay (Thermo, U.K.).

### 2.5 SDS PAGE and immunoblotting

Equal amounts of cytoplasmic protein lysate were mixed with 5× gel loading buffer (10% w/v SDS, 0.3 M TRIS-HCL, 25% v/v β-Mercaptoethanol and glycerol) and boiled for 10 min. The samples were then loaded on to pre-cast gradient (4–20%) acrylamide gels (BioRad, U.K.) and run for 2 h at constant 30 mA. After SDS PAGE, the separated protein was transferred to PVDF membrane using the BioRad midi system and Turbo Transblot (BioRad, U.K.). PVDF membranes were then blocked for 1 h in 5% w/v milk protein in TBS. Blocked membranes were then incubated with specific antibodies at 1:1000 dilution (p-PKR and PKR) or 1:5000 (Actin) in blocking buffer overnight at 4 °C with agitation. Detection of specific proteins was achieved by incubating the membranes in specific HRP conjugated secondary antibodies (eBioscience, U.K.) (1:2000 dilution in blocking buffer) for 1 h at room temperature. Membranes were washed 3 times in TBS 0.1v/v Tween and proteins detected using ECL (PerkinElmer, U.K.) and HyperFilm (Amersham, U.K.).

### 2.6 RNA extraction cDNA synthesis and qPCR

Total RNA was prepared as per the manufacturer's instructions (Bioline). For analysis of XBP1 splicing, total RNA was subjected to cDNA synthesis using Superscript cDNA synthesis kit (LifeTechnologies) as per the manufacturer's instructions. The resultant cDNA was then subjected to qPCR using SYBR Green (Anachem, U.K.) specific primers for spliced XBP1 (forward: 5′-TGCTGAGTCCGCAGCAGGTG-3′ reverse: 5′-GCTGGCAGGCTCTGGGGAAG-3′) and normalised to HPRT expression (forward: 5′-GACACTGGCAAAACAATG-3′ reverse: 5′-ACAAAGTCTGGCTTATATCC-3′). For CHOP and interferon-β expression, qRT-PCR was employed using commercial probe/primer sets (LifeTechnologies) and analysed using the Taqman ‘one-step’ system.

### 2.7 ELISA of interferon-β in BMDM supernatants

Wild type (PKR^+/+^) and PKR knock out (PKR^−/−^) BMDM were plated at 1 × 10^5^ cells per well of a 96-well plate. Cells were infected with *C. trachomatis* at an MOI of 20 for 24 h. Plates were centrifuged at 2000 × G for 5-min and the supernatants harvested. ELISA was performed on the supernatants to analyse interferon-β secretion using an in-house assay utilising capture antibody (monoclonal rat anti-mouse IFNβ IgG1; Santa Cruz: sc57201), detection antibody (polyclonal rabbit anti-mouse IFNβ; RnD Systems: 32400-1) and secondary antibody (goat anti-rabbit-HRP; Cell Signalling Technology 7074). Interferon standard curve was prepared using recombinant mouse interferon-β (Interferon Source; U.S.A).

### 2.8 Statistical analysis

Differences between multiple data sets were analysed using 1-Way ANOVA with Tukey's or Dunnet's post test correction where appropriate. Differences between two data sets were analysed using Student's *t*-test. Differences between wild type and knock out data sets were analysed using 2-Way ANOVA. *p* values of <0.05 were deemed significant.

## 3 Results

### 3.1 Agonists of pathogen recognition receptors or *Chlamydia* infection are potent activators of PKR in human mDC

**Fig. 1:**
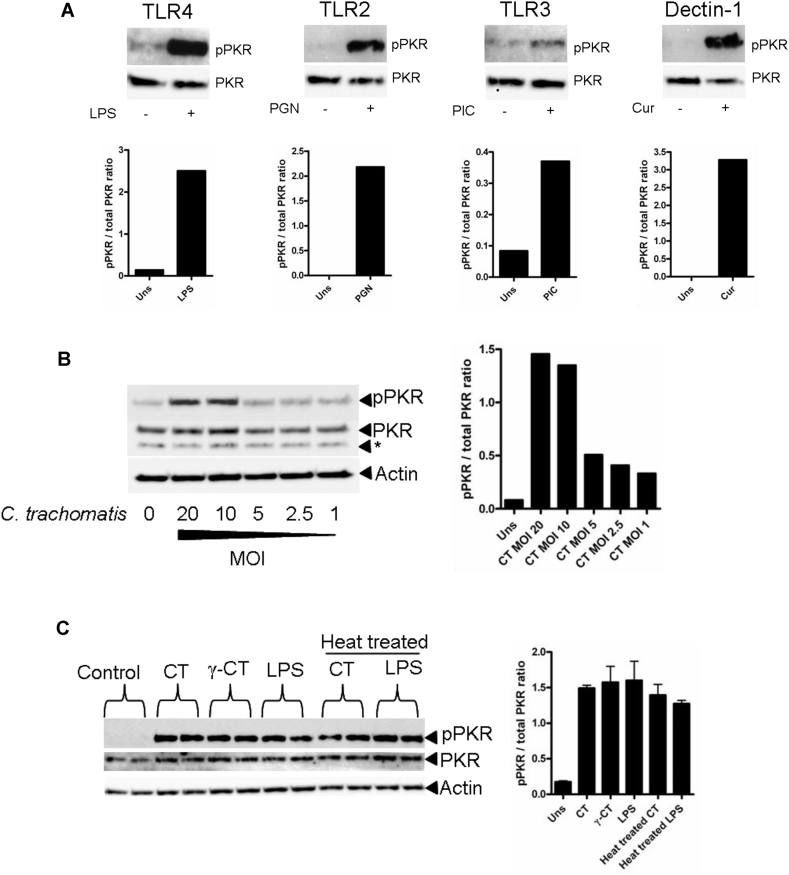
*Chlamydia trachomatis* induces PKR activation in mDC. (A) Western blot of PKR phosphorylation following stimulation with indicated PRR agonists for 4 h. Panel below indicates quantification by densitometry of the phosphorylated PKR band. (B) Western blot of PKR phosphorylation (pPKR) following infection with *C. trachomatis* for 8 h at different multiplicities of infection (MOI). (C) Western blot of PKR phosphorylation (pPKR) following stimulation with live *C. trachomatis* (CT), gamma ray-attenuated *C. trachomatis* (γ-CT), heat-treated *C. trachomatis*, LPS or heat-treated LPS for 8 h. Right panel indicates by densitometry of the phosphorylated PKR band.

Previous work demonstrated that TLR4 or TLR2 agonists are potent inducers of PKR phosphorylation in murine alveolar macrophages [5]. However, little is known about activation of PKR in primary human mDC, we therefore examined whether PKR activation occurred in response to stimulation of specific PRRs (Fig. 1A). Stimulation of mDC with agonists of TLR4 (LPS), TLR2 (Peptidoglycan), TLR3 (Poly I:C) or Dectin-1 (Curdlan) all induced a significant increase in PKR phosphorylation suggesting that PKR activation is a universal response to PRR ligation in mDC. We next investigated whether PKR is activated in response to infection with the intracellular bacterial pathogen *C. trachomatis*. To examine this, we infected human mDC at different multiplicities of infection (MOI) ranging from a ratio of 20 infectious units (IFU) per cell down to a ratio of 1:1 (Fig. 1B). We found that higher MOIs of 10–20 IFUs per cell induced the greatest increase in PKR phosphorylation compared to the non-infected control and that this was reduced at lower MOIs. We therefore conducted all future *Chlamydia* infection experiments at an MOI of 20. We next investigated whether intracellular replication of *C. trachomatis* was a requirement for PKR activation. To do this, we infected mDC with live, or heat-treated or gamma-irradiated attenuated *C. trachomatis* (which fail to replicate intracellularly in Hela cells), or stimulated cells with LPS or heat-treated LPS as a control (Fig. 1C). Both heat-treated and gamma-irradiated attenuated *C. trachomatis* were able to stimulate PKR activation in mDC to the same extent as live *C. trachomatis*, indicating that replication of *C. trachomatis* intracellularly, or the production by the *Chlamydiae* of a heat-labile pathogen associated molecular pattern (PAMP), were not responsible for the activation of PKR. Heat-treating LPS had no effect on its ability to activate PKR confirming its heat stability and suggests that *C. trachomatis* LPS is the likely PAMP required for PKR activation. It is unlikely to be *Chlamydia* hsp60, which has previously been implicated in TLR4 signalling during *Chlamydia* infection [13].

### 3.2 Chlamydia activates PKR through TLR4 and MyD88 signalling in contrast to *E. coli* LPS which required TLR4 and TRIF

**Fig. 2:**
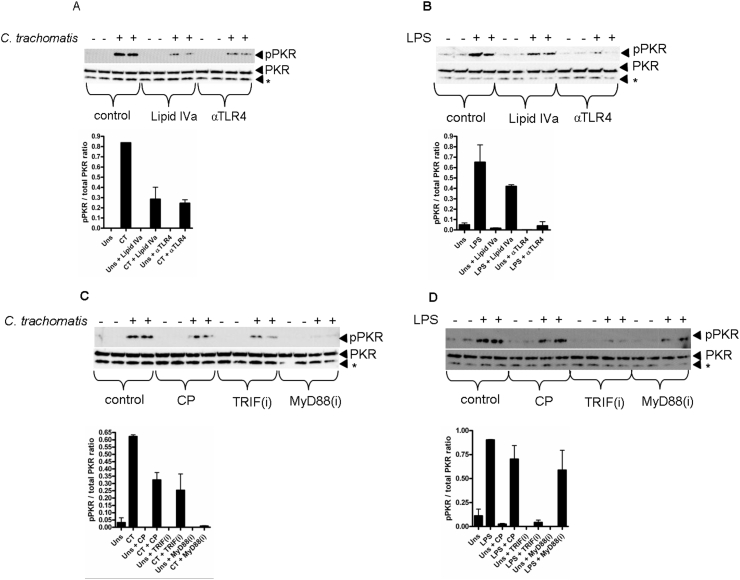
*Chlamydia trachomatis* induces PKR activation via TLR4 and MyD88 signalling. (A) Western blot of PKR phosphorylation (pPKR) following infection with *C. trachomatis* (MOI = 20) for 4 h in the presence of lipid IVa (1 μg/ml) or TLR4 blocking antibody (αTLR4) (10 μg/ml). (B) Western blot of PKR phosphorylation (pPKR) following LPS stimulation (1 μg/ml) for 4 h in the presence of lipid IVa (1 μg/ml) or TLR4 blocking antibody (αTLR4) (10 μg/ml). (C) Western blot of PKR phosphorylation (pPKR) following infection with *C. trachomatis* (MOI = 20) for 4 h in the presence of (50 μM) control peptide (CP), TRIF inhibitory peptide (TRIFi) or MyD88 inhibitory peptide (MyD88i). (D) Western blot of PKR (pPKR) phosphorylation following LPS stimulation (1 μg/ml) for 4 h in the presence of (50 μM) control peptide (CP), TRIF inhibitory peptide (TRIFi) or MyD88 inhibitory peptide (MyD88i). Panels below western blots indicate quantification by densitometry of the phosphorylated PKR band. * denotes non specific band.

*Chlamydiae* sp infection or stimulation with chlamydial heat shock proteins have previously been reported to activate TLR2 and TLR4 signalling to initiate inflammatory responses and cell death [12], [13], [14], [15], [16], [17]. *C. trachomatis* is a Gram negative organism and as such contains LPS in its outer membrane [18]; given that heat-killed *C. trachomatis* could induce PKR activation, suggesting that heat stable LPS might be responsible, we tested the hypothesis that TLR4 was the pathogen recognition receptor (PRR) required. To do this, we infected mDC with *C. trachomatis* (Fig. 2A) or, as a control, stimulated mDC with LPS (Fig. 2B), in the presence of a TLR4 blocking antibody or the TLR4 antagonist; lipid IVa. Blocking TLR4 signalling by either of these means potently inhibited *C. trachomatis*-induced PKR activation indicating a requirement for TLR4 in the induction of PKR activation in response to infection. TLR4 signalling can utilise two adaptor proteins, MyD88 and TRIF [19], [20], and a previous study demonstrated that activation of PKR in response to LPS was TRIF-dependent [6]. We therefore tested the hypothesis that *C. trachomatis*-induced PKR activation also required TRIF. To do this, we infected mDC with *C. trachomatis* (Fig. 2C) or, as a control, stimulated the cells with LPS (Fig. 2D), in the presence of MyD88 or TRIF inhibitory peptides, or a control peptide (CP). Compared to the control peptide (CP), LPS-induced PKR phosphorylation was entirely TRIF-dependent. Unexpectedly however, infection-induced PKR phosphorylation required MyD88 but was unaffected by TRIF inhibition compared to the control peptide (CP). Thus, there are distinct differences in the use of the adaptor proteins during *C. trachomatis* infection or stimulation with *E. coli*-derived LPS that contribute to PKR activation.

### 3.3 Infection induced PKR activation is independent of NADPH oxidase

**Fig. 3:**
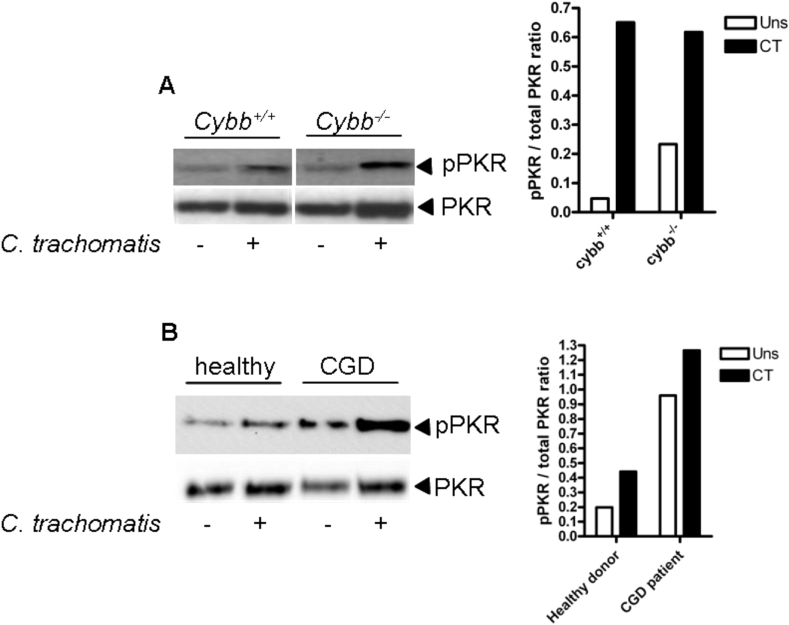
PKR activation is independent of NADPH oxidase. (A) Western blot of PKR phosphorylation (pPKR) in wild type (*cybb*^+/+^) or NADPH oxidase-deficient (*cybb*^−/−^) BMDM following infection with *C. trachomatis* for 8 h. Panel on the right indicates quantification by densitometry of the phosphorylated PKR band. (B) Western blot of PKR (pPKR) phosphorylation in mDC from a healthy donor or a CGD donor following infection with *C. trachomatis* for 6 h. Panel on the right indicates quantification by densitometry of the phosphorylated PKR band.

We next investigated what signals might be required in addition to TLR4 signalling to induce PKR activation. The mammalian NADPH oxidase system (NOX) is an important component of cellular host defence against microbial pathogens. Deletion or mutation of NOX2 results in immunodeficiency characterised by recurrent bacterial infection as observed in patients suffering with chronic granulomatous disease (CGD) [21], [22]. ROS signalling is known to be involved in many aspects of innate responses to microbes and sterile inflammation. Indeed, NOX-derived ROS have been implicated in the activation of PKR in response to cholesterol loading [9]. We therefore examined whether NADPH oxidase was required for PKR activation in response to infection in mDC and murine BMDM. Infection of BMDM (Fig. 3A) from wild type (*Cybb*^+/+^) or NOX deficient mice (*Cybb*^−/−^) or mDC (Fig. 3B) from healthy donors or CGD patients who lack a functional NADPH oxidase system, resulted in equivalent or even elevated (in the case of human mDC) PKR phosphorylation compared to healthy controls, indicating that NADPH oxidase is not required for *chlamydia* induced PKR activation and differs from cholesterol. However, we cannot rule out the possibility that ROS derived from other sources, such as the mitochondria, are involved in the activation of PKR in response to *C. trachomatis* infection.

### 3.4 The endoplasmic reticulum stress-inducing chemicals tunicamycin and thapsigargin induce PKR activation that is blocked by an inhibitor of IRE1α RNAse activity

**Fig. 4:**
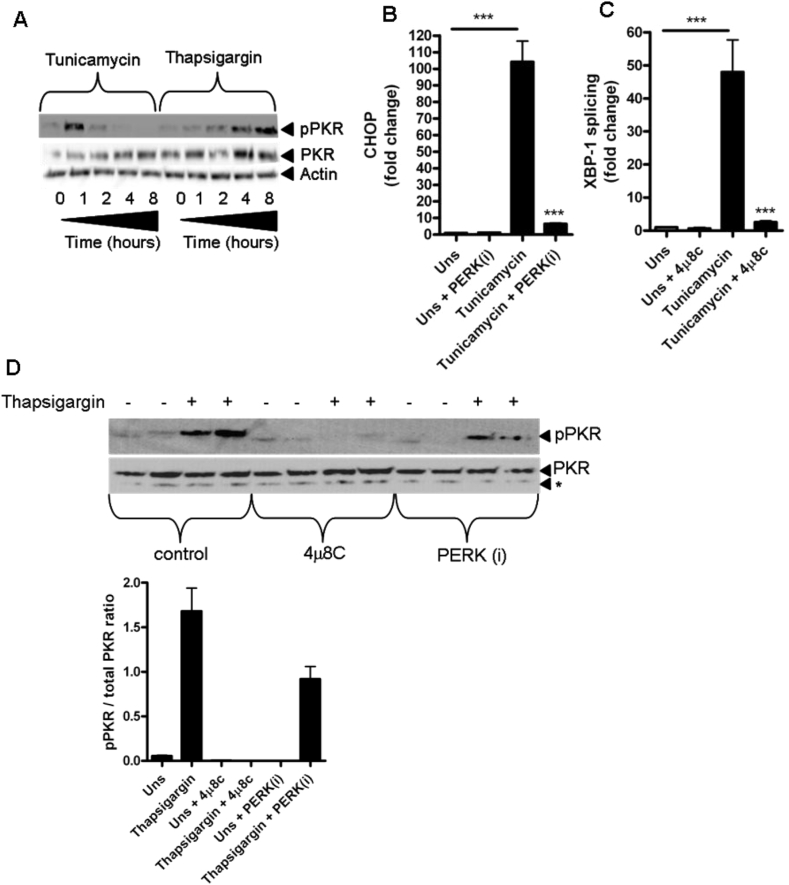
ER stress activates PKR that is blocked by an inhibitor of IRE1α RNAse activity. (A) Western blot of PKR phosphorylation (pPKR) in mDC following stimulation with Tunicamycin (1 μM) or Thapsigargin (0.25 μM) for indicated times. (B) CHOP mRNA expression in mDC following stimulation with tunicamycin (1 μM) for 4 h in the presence of GSK PERK inhibitor D3 (PERKi) (1 μM) *n* = 4 independent donors ***p = <0.001. Data represented as ±SEM. (C) XBP-1 splicing in mDC following stimulation with tunicamycin (1 μM) for 4 h in the presence of 4μ8C (30 μM) *n* = 4 independent donors ***p = <0.001. Data represented as ±SEM. (D) Western blot of PKR phosphorylation (pPKR) in mDC following stimulation with thapsigargin (0.25 μM) for 6 h in the presence of 4μ8C (30 μM) or GSK PERK inhibitor D3 (PERKi) (1 μM). Panel below indicates quantification by densitometry of the phosphorylated PKR band. * denotes non specific band.

The unfolded protein response (UPR) is a physiological mechanism that is initiated when the protein folding capacity of the ER is exceeded leading to ER stress [23]. Three ER sentinel proteins regulate the UPR: PERK, IRE1 and ATF6 which activate specific and shared target genes in response to ER stress resulting in either restoration of homeostasis or induction of cell death [23]. Additionally, activation of ER stress signalling pathways has been shown to be crucial for certain inflammatory responses resulting from TLR signalling and bacterial infections [12], [24], [25]. Significantly, ER stress-inducing agents are known to activate PKR [10], [11]. We therefore examined the hypothesis that ER stress signalling could activate PKR in mDC using chemical inducers of ER stress prior to examining the effects of *Chlamydia* infection. Stimulation of mDC with either tunicamycin or thapsigargin (Fig. 4A) resulted in potent phosphorylation of PKR, albeit with differing kinetics, confirming that ER stress leads to PKR activation. We next tested the hypothesis that the ER stress sentinels IRE1α or PERK regulated the mechanism of PKR activation. To do this, we utilised the well characterised inhibitors 4μ8c and GSK PERK inhibitor D3, which inhibit IRE1α RNAse activity and PERK activation respectively [26]. To confirm that 4μ8c and GSK PERK inhibitor D3 blocked the relevant ER stress pathways, we analysed CHOP expression (Fig. 4B) and XBP-1 splicing (Fig. 4C) as readouts of PERK or IRE1α RNAse activity respectively, in response to tunicamycin stimulation. As expected, stimulation of mDC with tunicamycin potently induced expression of CHOP and XBP-1 splicing that was almost entirely blocked by the specific inhibitors. We next examined PKR phosphorylation in response to thapsigargin stimulation in the presence of 4μ8c or GSK PERK inhibitor D3 (Fig. 4D). Interestingly PKR phosphorylation was completely blocked by 4μ8c but only partially by GSK PERK inhibitor D3, indicating that ER stress-induced PKR activation relied entirely on IRE1α RNAse activity and PERK activation to a lesser extent.

### 3.5 *C. trachomatis* infection of mDC induces TLR4-dependent and -independent ER stress responses

**Fig. 5:**
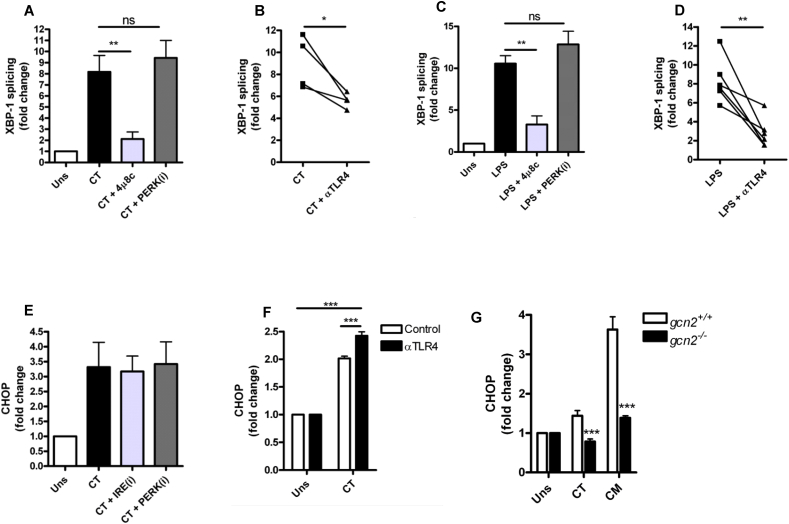
*Chlamydia* infection activates ER stress pathways that are dependent and independent of TLR4 signalling. (A) XBP-1 splicing in mDC following infection with *C. trachomatis* (CT) MOI = 20 for 4 h in the presence of 4μ8C (30 μM) or GSK PERK inhibitor D3 (PERKi) (1 μM) *n* = 4 independent donors **p = <0.01. Data represented as ±SEM. (B) XBP-1 splicing in mDC following infection with *C. trachomatis* (CT) MOI = 20 in the presence of a TLR4 blocking antibody (αTLR4) (10 μg/ml) *n* = 4 independent donors *p = <0.05. (C) XBP-1 splicing in mDC following LPS stimulation (1 μg/ml) for 4 h in the presence of 4μ8C (30 μM) or GSK PERK inhibitor D3 (PERKi) (1 μM) *n* = 4 independent donors **p = <0.01. Data represented as ±SEM. (D) XBP-1 splicing in mDC following LPS stimulation (1 μg/ml) for 4 h in the presence of a TLR4 blocking antibody (αTLR4) (10 μg/ml) *n* = 6 independent donors **p = <0.01. (E) CHOP mRNA expression in mDC following infection with *C. trachomatis* (CT) MOI = 20 for 4 h in the presence of 4μ8C (30 μM) or GSK PERK inhibitor D3 (PERKi) (1 μM) *n* = 4 independent donors. Data represented as ±SEM. (F) CHOP mRNA expression in mDC following infection with *C. trachomatis* (CT) MOI = 20 for 24 h in the presence of a TLR4 blocking antibody (αTLR4) (10 μg/ml). Data represented as ±SEM from 1 experiment performed in triplicate wells ***p = <0.001. (G) CHOP mRNA expression in wild type (*gcn2*^+/+^) or GCN2 knock out (*gcn2*^−/−^) MEFs following infection with *C. trachomatis* or *C. muridarum* (CM) MOI = 10 for 24 h. Data represented as ±SEM from 1 experiment performed in triplicate wells ***p = <0.001.

We have previously reported that *C. trachomatis* infection of mDC induces activation of the Integrated Stress Response (ISR) resulting in CHOP expression that enhances inflammatory responses [12]. However, there are no published data investigating IRE1α activation in response to *Chlamydia* infection. We therefore investigated XBP-1 splicing as an indicator of IRE1α activation in response to *C. trachomatis* infection (Fig. 5A). Infection induced robust XBP-1 splicing that was inhibited by 4μ8c but not by GSK PERK inhibitor D3 demonstrating that *C. trachomatis* infection was causing activation of IRE1α RNAse activity. Furthermore, we found that *Chlamydia* infection-induced IRE1α activation was dependent on TLR4 signalling as XBP-1 splicing was reduced in the presence of a TLR4 blocking antibody, and similar results were obtained with LPS as a control (Fig. 5C and D). We also confirmed that *C. trachomatis* infection induced CHOP expression in mDC, indicating activation of the ISR (Fig. 5E). Surprisingly, CHOP expression was independent of IRE1α and PERK activation as 4μ8c and GSK PERK inhibitor D3 had no effect on CHOP mRNA expression. Furthermore, *Chlamydia* infection-induced CHOP expression was independent of TLR4 signalling (Fig. 5F) as blocking TLR4 signalling with the TLR4 blocking antibody, resulted in *increased* CHOP expression in response to *C. trachomatis* suggesting that induction of the ISR occurs independently of LPS and TLR4 and is therefore distinct to the activation of IRE1α and PKR. Mammalian cells also express two additional eIF2α kinases, namely GCN2 and HRI, which are activated in response to amino acid or heme depletion respectively [27]. *Chlamydiae* sp have been termed ‘energy parasites’ as they utilise host cell metabolites such as amino acids [28]. Given that we have provided evidence that CHOP induction was independent of PERK and TLR4 induced PKR activation, we tested the hypothesis that CHOP induction in response to *Chlamydia* infection occurs through activation of the amino acid responsive eIF2α kinase GCN2. To do this, we infected wild type (*gcn2*^+/+^) or GCN2 knock out (*gcn2*^−/−^) MEFs with *C. trachomatis* or the murine pathogen *Chlamydia muridarum* (that induces a more potent CHOP response than *C. trachomatis* in MEFs) and examined CHOP expression (Fig. 5G). Interestingly, induction of CHOP expression by *C. trachomatis* or *C. muridarum* infection was entirely GCN2 dependent indicating that although infection resulted in PKR activation, GCN2 is the likely eIF2α kinase responsible for the induction of the ISR and is independent of IRE1α, PKR, PERK and TLR4 signalling.

### 3.6 TLR4/IRE1α signalling mediates PKR activation and is required for enhancement of type-1 interferon in response to *C. trachomatis* infection

**Fig. 6:**
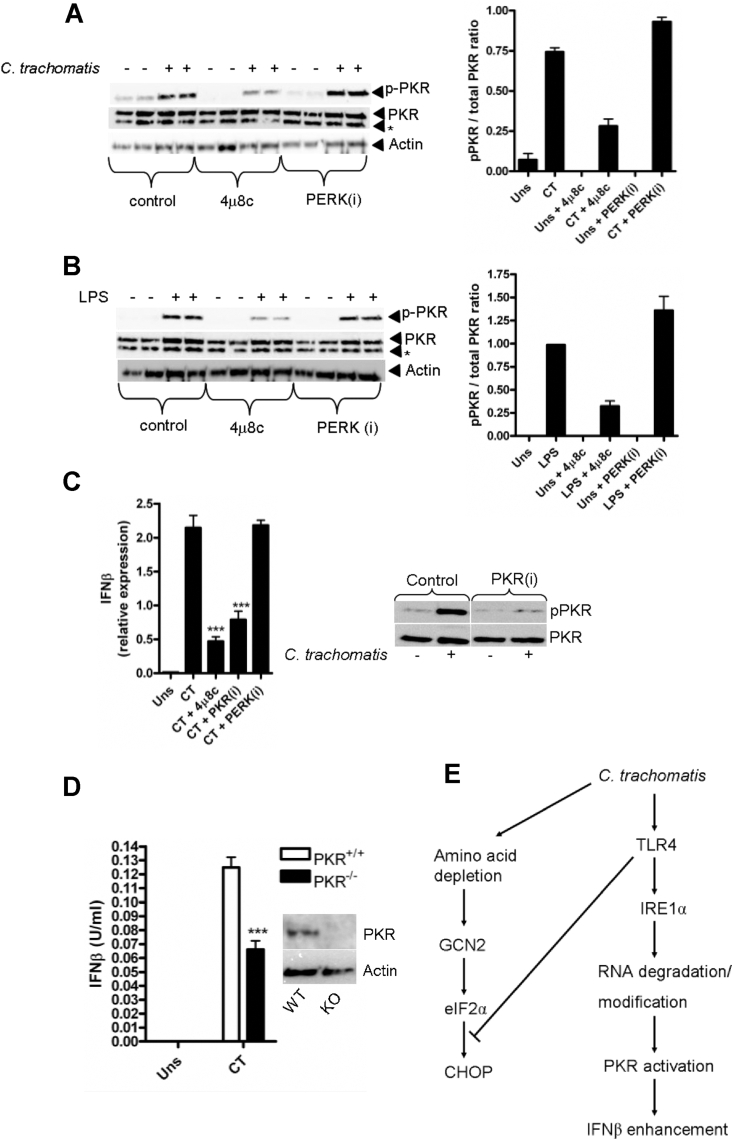
TLR4 induced PKR activation is blocked by an inhibitor of IRE1α RNAse activity but not PERK phosphorylation and is required for enhancement of Interferon-β production. (A) Western blot of PKR phosphorylation (pPKR) following infection with *C. trachomatis* (MOI = 20) for 4 h in the presence of 4μ8C (30 μM) or GSK PERK inhibitor D3 (PERKi) (1 μM). Panel on the right indicates quantification by densitometry of the phosphorylated PKR band. (B) Western blot of PKR phosphorylation (pPKR) following LPS stimulation (1 μg/ml) for 4 h in the presence of 4μ8C (30 μM) or GSK PERK inhibitor D3 (PERKi) (1 μM). * denotes non-specific band. Panel on the right indicates quantification by densitometry of the phosphorylated PKR band. (C) Interferon-β mRNA expression in mDC following infection with *C. trachomatis* (CT) MOI = 20 for 8 h in the presence of 4μ8C (30 μM), PKR inhibitor C16 (PKRi) (500 nM) or the GSK PERK inhibitor D3 (PERKi) (1 μM). Data represented as ±SEM from 1 experiment performed in triplicate wells ***p = <0.001. Right panel depicts western blot of PKR phosphorylation (pPKR) in mDC in response to *C. trachomatis* infection for 4 h in the presence of the PKR inhibitor C16 (PKRi) (500 nM). (D) ELISA of interferon-β secretion (U/ml) in supernatants from wild type (PKR^+/+^) or PKR knock-out (PKR^−/−^) BMDM infected with *C. trachomatis* (CT) MOI = 20 for 24 h. Data represented as ±SEM from 1 experiment performed using BMDM obtained from three separate individual wild type or knock out mice ***p = <0.001. Right panel depicts western blot of PKR expression in wild type (WT) or PKR knock out (KO) BMDM. (E) Schematic representation of pathways activated in response to *C. trachomatis* infection.

We have demonstrated that ER stress induced PKR activation was inhibited by 4μ8C suggesting that PKR activation in response to ER-stress requires IRE1α RNAse activity. Furthermore, we have shown that *C. trachomatis* infection or LPS stimulation resulted in potent PKR phosphorylation that was TLR4 dependent and independent of NADPH oxidase. Lastly, we provided evidence that infection or LPS stimulation results in the activation of IRE1α that is also TLR4 dependent. We therefore tested the hypothesis that infection- and LPS-induced PKR phosphorylation occurs as a consequence of IRE1α RNAse activity. To do this we infected mDC with *C. trachomatis* (Fig. 6A) or stimulated with LPS (Fig. 6B) in the presence of 4μ8c or GSK PERK inhibitor D3. Importantly 4μ8c, but not GSK PERK inhibitor D3, potently blocked both *C. trachomatis*- and LPS-induced PKR activation. These data are suggestive of a novel, universal mechanism for the activation of PKR during non-viral infection, in the absence of viral dsRNA, such as occurs during bacterial infection. Finally, we wished to address a role for PKR during *Chlamydia* infection. Previous reports have demonstrated that PKR activation in response to TLR4 stimulation is required for the enhancement of type-1 interferon production [6]. Given that we observed an apparent redundancy for PKR in the activation of the integrated stress response, we hypothesised that PKR may play an alternative inflammatory role in response to *Chlmaydia* infection. Importantly, 4μ8c and the specific PKR inhibitor-C16 (PKRi), significantly reduced interferon-β transcription in mDC, while the PERK inhibitor (that did not affect PKR activation) had no effect (Fig. 6C). This suggests that TLR4/IRE1α mediated PKR activation enhances type-1 interferon response following *Chlamydia* infection and indicates that the role of PKR during infection is one of regulating inflammatory, rather than translational responses. To confirm our results in human mDC, we infected PKR wild type and PKR knock-out BMDM with *C. trachomatis* and analysed interferon-β secretion (Fig. 6D). Crucially, PKR deficient BMDM showed reduced interferon secretion in response to infection than the wild-type cells reinforcing our data using mDC.

## 4 Conclusions

In this study we have demonstrated that infection of monocyte-derived DC with *C. trachomatis* or stimulation with LPS results in TLR4-dependent activation of the IRE1α branch of the UPR, and that an inhibitor of IRE1α RNAse activity blocks PKR phosphorylation. Furthermore, inducing ER stress in mDC also resulted in PKR phosphorylation that was dependent on IRE1α RNAse activity. Taken together, these data suggest a universal mechanism of PKR activation by TLR signalling in the absence of dsRNA. A possible explanation for the central role for IRE1α is that host mRNAs, processed by IRE1α-through Regulated IRE1α Dependent Decay (RIDD) [29], may provide RNA structures that are recognised by PKR as damage associated molecular patterns (DAMPs). In support of this hypothesis, RIDD processed mRNA can act as a DAMP by activating the cytosolic PRR, RIG-I [30]. It is therefore tempting to speculate that a similar process may occur during *C. trachomatis* infection resulting in PKR activation through detection of host degraded mRNA, possibly through interactions with RIG-I. Alternatively, a recent report has suggested that small nucleolar RNA (snoRNA) are capable of activating PKR in response to metabolic stress induced by palmitic acid [31]. Furthermore, it has been demonstrated that PKR phosphorylation in response to thapsigargin or palmitic acid stimulation relies upon a functional dsRNA binding domain in PKR [10]. Again, we suggest these previous findings support our hypothesis that PKR activation in response to TLR4 stimulation or infection is occurring through detection of host RNA species that are induced or modified through IRE1α RNAse activity. In further support of this hypothesis, the inhibitor 4μ8C, does not affect the kinase activity of IRE1α, but functions by forming a Schiff base with a critical lysine residue within the endonuclease domain of IRE1α [32].

Interestingly, we found that *C. trachomatis* induced PKR activation utilised MyD88 as an adaptor while we found in accordance with other reports, that *E. coli* derived LPS utilised TRIF [6]. This finding is surprising given that *C. trachomatis* is an intracellular pathogen and MyD88 signalling is thought to integrate TLR4 signals originating from the plasma membrane, while TRIF is utilised by TLR4 signalling from endosomal compartments [33]. This suggests that MyD88 may have a role during intracellular bacterial infection signalling from endosomal compartments leading to PKR activation. A further explanation for the difference in adaptor use between *C. trachomatis* and *E. coli* LPS is the structure of the lipid A moieties. Lipid A from *C. trachomatis* is penta-acylated while *E. coli* lipid A is hexa-acylated. Recent evidence has demonstrated that in comparison to hexa-acylated LPS, penta-acylated LPS induces weak TLR4 signalling as it does not induce TLR4 dimerisation and endocytosis. Furthermore, penta-acylated LPS can inhibit hexa-acylated LPS induced TRIF responses but maintain myddosome formation [34]. This finding may explain TLR4 reliance on MyD88 and not TRIF as an adaptor in response to *C. trachomatis* infection to induce PKR activation. To our knowledge there are no published studies investigating the lipid A acylation status of LPS and PKR activation. Studies have shown that the acylation status of lipid A is crucial for determining activation of inflammatory responses [35], therefore investigating whether acylation status of lipid A determines adaptor usage during TLR4 induced PKR activation would be worthwhile. Importantly, other reports have demonstrated MyD88 signalling is the predominant adaptor protein involved in *Chlamydia* species-induced inflammatory responses and our data compliment these previous findings [36], [37].

Infection induced PKR activation did not require NADPH oxidase, in contrast to cholesterol loading induced PKR activation [9]. However, other cellular sources of ROS have been identified, notably mitochondrial-derived ROS that have been demonstrated to be a key component of the innate inflammatory response in myeloid cells [38], [39]. Furthermore, mitochondrial derived ROS have been implicated in PKR activation [7]. We have demonstrated that NADPH oxidase and presumably, NADPH oxidase derived ROS are dispensable for *Chlamydia* induced PKR activation. However, we cannot entirely rule out a role for ROS produced from alternate sources such as the mitochondria.

We also report the interesting, but paradoxical observation that activation of the integrated stress response (ISR) resulting in CHOP expression as a consequence of *Chlamydia* infection was independent of TLR4 and by extension, the eIF2α kinase PKR. This observation is supported by evidence that demonstrates that TLR4 signalling actually suppresses activation of the ISR [40]. However, despite TLR4 suppression, *C. trachomatis* infection still resulted in CHOP expression, indicating activity of another eIF2α kinase that was distinct from TLR4/IRE1α mediated PKR activation. Significantly, inhibition of PERK also failed to prevent *Chlamydia*-induced CHOP expression, indicating that another eIF2α kinase distinct from PERK or PKR was responsible. Mammalian cells possess the eIF2α kinases GCN2 and HRI in addition to PERK and PKR. GCN2 responds to amino acid starvation and represents a highly conserved mechanism of nutrient sensing. Using GCN2 deficient MEFs we have demonstrated that induction of the ISR by *Chlamydia* infection was dependent on GCN2, suggesting that *Chlamydiae* induce an amino acid-deprived state within the infected host cell. *Chlamydiae* sp are known to utilise host cell amino acids [41], [42] and this could potentially lead to depletion of intracellular amino acid levels leading to GCN2 activation. However, given that our experiments were carried out using cell growth medium that has excess concentrations of amino acids, Chlamydial depletion of host amino acids through metabolism appears unlikely. An alternative possibility is suggested by the observation that intracellular infection with *Shigella flexneri* induces host cell membrane damage resulting in activation of GCN2 through amino acid depletion via an undefined mechanism [43]. *Chlamydiae* replicate intracellularly within a membrane bound parasitophorous vacuole termed the inclusion [44]. Recent work has demonstrated that the inclusion membrane is attacked during infection by host GTPases leading to membrane damage and the induction of antimicrobial autophagy responses [45]. Therefore, GTPase-induced membrane damage during *Chlamydia* infection could lead to GCN2 responses via a similar mechanism to that identified during *Shigella* infection. A further possibility is that *Chlamydia* infection results in reduced tryptophan concentrations intracellularly, as a consequence of catabolic metabolism of tryptophan by the inducible enzyme; Indoleamine 2,3-dioxygenase (IDO) [46]. Reduced tryptophan concentrations secondary to host responses could therefore drive activation of GCN2 and the ISR. We have previously demonstrated a pro-inflammatory role for CHOP during *C. trachomatis* infection, enhancing IL-23 production; this required live, replicating *Chlamydia* [12]. Thus the role of GCN2 responses in the induction of CHOP and its consequences for cytokine responses represents an intriguing line of enquiry. Additionally, further investigation is required to understand why PKR-despite it being potently activation by TLR4 signalling, is not required for ISR activation through its eIF2α kinase activity? We have provided evidence that PKR contributes to the enhancement of inflammatory responses as a consequence of TLR4 activation and suggests a potential dual role for PKR as either an eIF2α kinase or an inflammatory mediator depending on its activatory signal.

Finally, using PKR deficient BMDM and inhibitors which block PKR activation, we have demonstrated that PKR activation contributes to type-1 interferon production in response to *C. trachomatis* infection. PKR has previously been reported to contribute to the induction of interferon-β transcription during TLR4 stimulation of macrophages [6] and our findings with *Chlamydia* infection are in agreement with this. Crucially, we also find that 4μ8c which blocked PKR activation in mDC, also reduced transcription of interferon-β to a similar extent as the specific PKR inhibitor C16 (PKRi) thereby reinforcing our findings that IRE-1α RNAse activity contributes to PKR activation and subsequent PKR mediated responses.

In summary we have demonstrated a novel mechanism of PKR activation in response to *Chlamydia* infection, which requires TLR4 and IRE1α and that PKR enhances inflammatory responses. We have also demonstrated that activation of the ISR following *Chlamydia* infection occurs through the eIF2α kinase GCN2, presumably due to reduced amino acid availability, and is independent of TLR4, IRE1α, PKR and PERK. We therefore suggest that TLR4 activation of IRE1α RNAse activity, results in the production of modified host RNA species which are detected by PKR, leading to its activation. These data provide an attractive explanation for the activation of PKR during bacterial infections in the absence of viral dsRNA.

## Conflict of interest

All authors confirm that there are no conflicts of interest.
